# Adrenal-derived factors drive progression of sclerotic prostate cancer in bone

**DOI:** 10.1530/ERC-25-0309

**Published:** 2026-06-09

**Authors:** Malin Hagberg Thulin, Lei Li, Sanna Abrahamsson, Andreas Landin, Karin Horkeby, Jianyao Wu, Marie K Lagerquist, Claes Ohlsson, Matti Poutanen

**Affiliations:** ^1^ Department of Internal Medicine and Clinical Nutrition, Institute of Medicine, Sahlgrenska Osteoporosis Centre, Centre for Bone and Arthritis Research at The Sahlgrenska Academy, University of Gothenburg, Gothenburg, Sweden; ^2^ Bioinformatics and Data Centre, Sahlgrenska Academy, University of Gothenburg, Gothenburg, Sweden; ^3^ Department of Clinical Chemistry, Sahlgrenska University Hospital, Gothenburg, Sweden; ^4^ Research Centre for Integrative Physiology and Pharmacology, Institute of Biomedicine and Turku Center for Disease Modeling, University of Turku, Turku, Finland; ^5^ FICAN West Cancer Center, University of Turku and Turku University Hospital, Turku, Finland

**Keywords:** castration-resistant prostate cancer, adrenal glands, sex hormones, dihydrotestosterone, bone metastasis, sclerotic bone, osteogenesis, angiogenesis

## Abstract

Bone metastasis is a leading cause of death in prostate cancer (PC) patients. Although androgen deprivation therapy (ADT) combined with novel androgen‐targeted agents constitutes the cornerstone of systemic treatment, its efficacy is limited. We investigated the adrenal contribution to promoting progression of castration-resistant PC (CRPC) within bone using a preclinical intratibial xenograft model (VCaP, 22Rv1, and LNCaP cells). Mice underwent orchiectomy (ORX) to mimic ADT, with or without adrenalectomy (ORX + ADX) to eliminate adrenal contribution. A significant increase in bone mineral density (BMD) was observed in tumor-grafted tibiae in ORX-treated mice compared with controls (*P* < 0.001), indicating a strong tumor-induced sclerotic response. In contrast, ORX + ADX reduced tumor take rate by approximately 50% and decreased tumor-induced BMD by over 80% (*P* < 0.001). Transcriptomic analysis revealed that ADX downregulated tumor-induced transcripts in bone by over 90%, including osteogenic (*Lox, Sparcl1, Bmp2, Postn*, and *Col1a1*) and pro-angiogenic (*Bmper, Pecam-1,* and *Esam*) signatures. In addition, BMP, PI3K/Akt, and ERK1/2 signaling pathways were associated with the tumor-induced bone response. Both high serum progesterone and intratumoral levels of dihydrotestosterone (DHT) were associated with the sclerotic bone phenotype. ADX markedly reduced intratumoral DHT and downregulated glycolytic genes (*HK2, PFK2,* and *LDHA)* and secretory proteins expressed by the tumor, including stanniocalcin 2, potentially mediating paracrine effects in the sclerotic bone response. Altogether, these findings highlight the critical role of adrenal-dependent androgen synthesis, particularly via progesterone, in driving the sclerotic CRPC in bone. Our findings suggest that a comprehensive blockade of adrenal contribution is essential to prevent the sclerotic bone response associated with CRPC.

## Introduction

Bone metastasis is the leading cause of death in prostate cancer (PC) patients (1, 2). Over 90% of individuals diagnosed with advanced PC display indications of skeletal metastases, which are typically sclerotic, making the management of these lesions a major clinical challenge (3, 4). Therefore, understanding the reciprocal interaction between tumor cells and the bone microenvironment and its regulation is crucial for developing more effective treatments.

Androgen deprivation therapy (ADT), androgen receptor (AR) signaling inhibition, and chemotherapy dominate the medical treatments in recurrent and metastatic PC (5). Despite these treatments, patients eventually develop castration-resistant PC (CRPC), which has a poorer prognosis and remains largely driven by androgens (6, 7). ADT lowers circulating testosterone levels by more than 90% by suppressing testicular production, and the remaining 5–10% of testosterone is believed to originate from peripheral conversion of adrenal-derived precursors (8, 9, 10, 11). To encounter this issue, therapies targeting both AR and androgen synthesis pathways, such as enzalutamide and abiraterone, have been developed (12, 13). These therapies are now established as first-line medical treatments for metastatic hormone-sensitive prostate cancer (mHSPC) and metastatic CRPC (mCRPC) (13, 14, 15, 16, 17). Radiographic progression-free survival (rPFS) is strongly associated with overall survival (OS) in CRPC (18, 19), and both enzalutamide and abiraterone have significantly enhanced OS and rPFS in CRPC patients with bone metastases (16, 19, 20, 21). Nonetheless, therapeutic efficacy remains limited, and treatment resistance continues to represent a significant clinical challenge.

The significant role of adrenals in advancing CRPC is highlighted in historical reports, demonstrating that adrenalectomy (ADX) delayed disease relapse and improved radiographic regression of sclerotic bone metastases (22, 23, 24). This underscores the critical role of adrenal-dependent signaling in the sclerotic progression of bone lesions in PC. However, emerging evidence suggests that factors beyond AR-mediated pathways may also contribute to the lethal progression of CRPC within the bone microenvironment (25, 26, 27).

In this study, we investigated the role of adrenal regulation in the sclerotic progression of CRPC in bone using an intratibial xenograft model that closely mimics the clinical scenario. Because residual androgen-derived steroid precursors persist after ADT and can sustain AR signaling and tumor progression, we compared orchiectomy (ORX) alone with combined ORX and adrenalectomy (ORX + ADX) to identify adrenal-dependent mechanisms driving bone metastatic progression in CRPC.

## Methods

### Intratibial *in vivo* model

Male Balb/c nude male mice (Janvier, France), 7–8 weeks old, were allowed to acclimatize for at least 7 days before initiating the experiments. The mice were housed in individually ventilated cages and had free access to chow pellets (Teklad Global 16% diet, Envigo–Harlan, USA) and tap water in pathogen-free conditions.

For the establishment of the sclerotic model for intratibial CRPC xenografts, we used three different AR-positive human PC cell lines (VCaP, 22Rv1, and LNCaP). The cells were obtained from the American Type Culture Collection (ATCC, USA) and cultured *in vitro* according to the instructions of the provider, using cell culture reagents purchased from Gibco™, Thermo Fisher Scientific (USA). One million cells resuspended in 7 μL of Matrigel (BD Biosciences, USA) were injected into the bone marrow cavity of the right tibia of 9- to 11-week-old Balb/c nude males. The contralateral left tibia was injected with Matrigel only and served as an internal control for the tumor-induced response. To mimic clinical CRPC and investigate adrenal contribution to the sclerotic bone progression, mice were subjected to bilateral orchiectomy (ORX), orchiectomy combined with bilateral adrenalectomy (ORX + ADX), or sham-operated at the time of tumor cell implantation. After ADX, the drinking water was replaced with 0.9% NaCl. The mice were anesthetized with isoflurane inhalation (IsoFlo Vet, Abbott Laboratories, Netherlands) during surgery. Analgesia was provided by Metacam (0.5 mg/mL, Boehringer Ingelheim Animal Health, Germany) once before surgery and for 3 days postoperatively. Blood samples were collected from the saphenous vein via cardiac puncture at sacrifice. For histology, tibiae were excised and fixed in 4% neutral buffered formalin for 48 h, followed by fixation in 70% ethanol. Subsequently, bones were decalcified in EDTA and embedded in paraffin. For RNA extraction and steroid measurements, bones were snap-frozen in liquid nitrogen and stored at −80°C until use.

### Dual-energy X-ray absorptiometry

To monitor and determine tumor-induced bone formation, the tumor-injected tibia and the contralateral sham-injected tibia from each mouse were analyzed *in vivo* using repeated X-ray analyses (Faxitron Bioptics, USA) with an isotropic pixel size of 48 μm. A defined region of interest (ROI), starting from the proximal growth plate to the distal tibiofibular joint, corresponding to ∼20% of the total length of the tibia, was analyzed. The ROI area was determined using the ruler-tool software provided with the scanner. Tumor-induced bone formation was determined by calculating the difference in bone mineral density (BMD; mg/cm^2^) or the BMD ratio between the tumor-injected and the sham-operated tibia.

### 3D imaging by μCT

High-resolution micro-computed tomography (μCT) was used to analyze the bone structure of the tumor and control tibia (Skyscan, 1275; Bruker MicroCT, Belgium). The bones were imaged with an X-ray tube voltage of 40 kV, using a current of 200 μA, and a 1 mm aluminum filter. The scanning angular rotation was 180°, and the angular increment was 0.40°. The voxel size was 7 μm isotropically. NRecon (Bruker) was employed to perform the reconstruction after the scans.

### Steroid analysis

Tumor bones and sham-treated bones were thawed on ice; a defined length with a start from the proximal growth plate to the distal tibiofibular joint, corresponding to ∼20% of the total length of the tibia, was cut into 1 mm pieces, weighed, placed in 2 mL Eppendorf tubes with 450 μL phosphate-buffered saline, and homogenized by shaking (30 Hz) with a 5 mm steel bead in a Tissuelyzer for 2 × 5 min. Serum samples were measured volumetrically to 200 μL by pipetting. For calculation purposes, analyte values below the lower limit of quantification (LLOQ) were set at half the LLOQ specific to that analyte (28, 29). Sex steroid extraction, derivatization, and measurement by gas chromatography–tandem mass spectrometry (GC–MS/MS) were performed as previously reported. Detailed assay validation, including the sensitivity of the assay in various types of matrices, has been previously published (29).

### Human prostate-specific antigen EIA

Human prostate-specific antigen (PSA) in serum from ORX and ORX + ADX mice was measured by an immunoenzymetric assay (CanAg PSA EIA; 340–10, Fujirebio Diagnostics, Sweden) with a minimum detectable value of <0.1 μg/L.

### RNA sequencing

Total mRNA was extracted from snap-frozen bones with the intratibial VCaP tumors from ORX and ORX + ADX mice and their corresponding contralateral non-tumor-bearing control tibia (*n* = 8 mice from each group, respectively) using TRIzol reagent (Thermo Fisher Scientific) followed by the RNeasy Mini Kit (Qiagen, Germany). The quantity (ng/μL) and purity (260/280 nm) of the extracted RNA were measured spectrophotometrically using the IMPLEN NanoPhotometer® (Germany). RNA sequencing was then performed by Novogene Europe (UK), which applied their standard pipeline.

#### *Preprocessing*

The raw reads were quality-filtered by removing reads with adapter contamination, reads with >10% unknown bases (Ns), and reads with <5 Q-scores in more than 50% of bases. The quality-filtered reads were then aligned to the reference genomes (human: GRCh38 and mouse: GRCm38) using HISAT2. The read counts were recorded using the ENSEMBL annotations.

#### *Differential expression*

The raw counts of the human and mouse annotations were separately loaded into the R package 4.1.3. Four samples of the ORX + ADX mice were removed due to low counts of human reads (5,000–15,000 counts). Genes with zero counts were also removed. The R package DESeq2 (v. 1.34.0) was used for the differential expression analysis (30) by estimating the size factors and the dispersion, fitting a negative binomial generalized linear model (GLM), and calculating the statistics using Wald statistic. The *P*-values were corrected using the Benjamini–Hochberg approach. The log2 fold estimates were shrunk using the normal method (30). The counts were transformed using regularized log^2^. The R package pheatmap (v. 1.0.12) was used to generate heatmaps using the transformed counts with the subtracted mean. The plotted genes were clustered using hierarchical clustering.

Significantly altered transcripts (adj. *P* < 0.01 for mouse and adj. *P* < 0.05 for human) between ORX tumor vs ORX sham and ORX + ADX tumor vs ORX + ADX sham tumors (human) and bone (mouse) were extracted and were separated into those with positive and negative log2 fold changes (FCs). Enrichment analyses for Gene Ontologies (31) and KEGG pathways (32) were generated in R (v. 4.3.0) (33) using clusterProfiler (v. 4.8.1 for mouse transcripts and v. 4.22 for human). A Venn diagram was generated using ggven (v. 0.1.9) to visualize the overlap between the groups. For the human transcripts, EnhancedVolcano (v. 1.12.0) was used with a cutoff adj. *P*-value of <0.05 on the y-axis and a log2 FC cutoff of 1 on the x-axis.

We also generated a list (2) of AR-associated genes containing known androgen-regulated genes as well as AR-interacting proteins from different resources, including previous publications (34); database resources of the National Center for Biotechnology Information (35); the androgen pathway product list provided by SwitchGear Genomics (USA); and the Regulators of Androgen Action Resource (RAAR) database (36). The significantly altered transcripts (adj. *P* < 0.05) between the ORX tumors and ORX + ADX tumors that showed overlap with AR-associated genes were extracted. The transformed counts with the subtracted means were visualized in a heatmap generated using pheatmap (v.1.0.12) using hierarchical clustering.

#### Correlation

Normalized counts (TPMs) of the transcripts from the ORX tumor vs ORX sham (mouse) and ORX tumor vs ORX + ADX tumor (human) were extracted and correlated with the serum levels of PSA, DHT, progesterone, and testosterone using Spearman correlation. The *P*-values were adjusted using the Benjamini–Hochberg approach.

#### String analysis

To predict functional protein networks of the tumor-induced osteogenic signature in ORX mice, STRING version 12.0 (37) was used with a confidence score = 0.4. Clustering was performed using the Markov cluster algorithm (MCL), with default inflation parameters, to identify natural clusters based on the stochastic flow.

### Statistics

A paired Student’s *t*-test was used to evaluate the effect of tumors on BMD in ORX mice. To assess statistical differences across groups in ORX mice, a two-way ANOVA was performed on changes in BMD, followed by Sidak’s post hoc test to correct for multiple comparisons. For comparisons of mouse-related data among all groups (ORX sham, ORX tumor, ORX + ADX sham, and ORX + ADX tumor), Kruskal–Wallis H tests were employed. Human data comparisons between ORX tumor and ORX + ADX tumor groups were analyzed using the Mann–Whitney U test or one-way ANOVA, as appropriate. Spearman correlation was conducted to explore the relationships between sex steroid tissue levels in bone lesions, tumor-induced BMD, and serum PSA. In addition, a chi-square test was performed to determine the significant association between PSA and tumor take rate. GraphPad Prism 9 (GraphPad, USA) was used for all statistical analyses. The significance levels were set at *P* < 0.05 (*), *P* < 0.01 (**), *P* < 0.001 (***), and *P* < 0.0001 (****).

## Results

### Adrenal-derived factors are essential for tumor-induced sclerotic bone response in castrated mice

**Figure 1:**
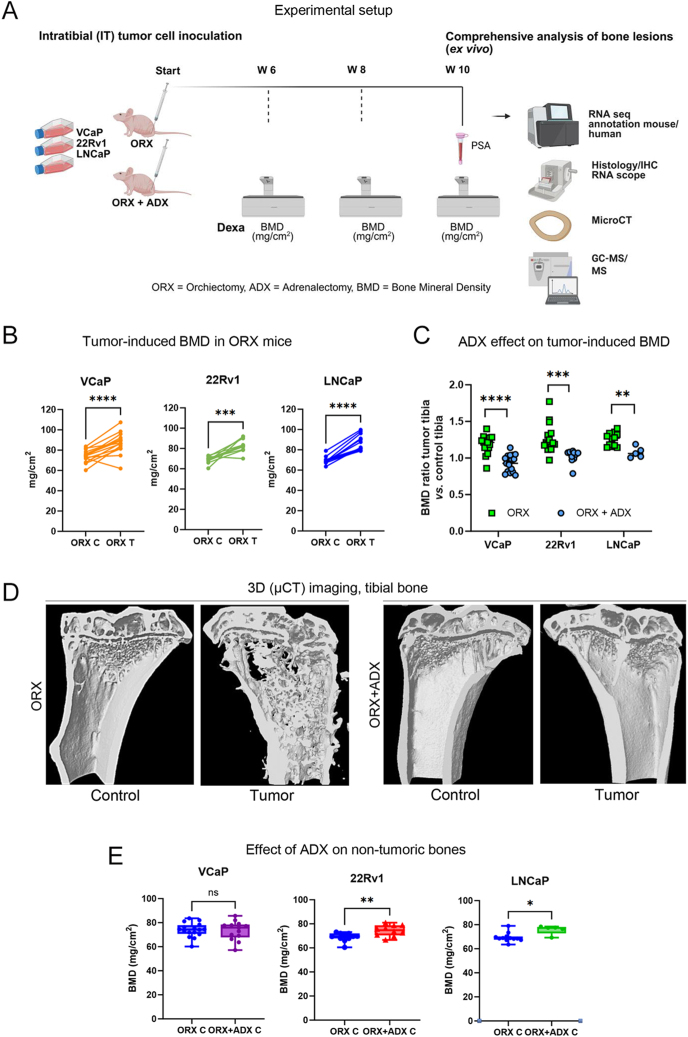
Tumor-induced bone response in orchiectomized (ORX) and orchiectomized plus adrenalectomized (ORX + ADX) mice at termination. (A) Experimental setup. (B) Bone mineral density (BMD), measured by dual-energy X-ray (DEXA), of the tibia in ORX mice injected with VCaP, 22Rv1, and LNCaP tumor cells (T) and sham-injected control tibia (C) of the same mice. (C) Ratio of the BMD measured in the tumor cell-injected tibia (T) to the sham-injected control tibia (C) in ORX and ORX + ADX mice injected with VCaP, 22Rv1, and LNCaP tumor cells. ORX mice are indicated by green squares, and ORX + ADX mice are indicated by blue dots. (D) Representative μCT images of the sham-injected tibia (control) and VCaP-injected tibia (tumor) in ORX and ORX + ADX mice. (E) Measured BMD in sham-injected control bones (C) in ORX and ORX + ADX mice. A paired Student’s *t*-test was applied to determine tumor-induced BMD in ORX mice (B), a Kruskal–Wallis H test was used to determine differences in tumor-induced BMD between ORX and ORX + ADX mice (C), and a Mann–Whitney U test was used to assess differences in BMD in sham-injected ORX and ORX + ADX mice (E). *P* < 0.05 (*), *P* < 0.01 (**), *P* < 0.001 (***) and *P* < 0.0001 (****).

To model the growth of sclerotic CRPC lesions in the bone, we used three human PC cell lines (VCaP, 22Rv1, and LNCaP) that were inoculated in the bone marrow cavity of the tibia of castrated (ORX) mice (Fig. 1A). All three models exhibited a robust sclerotic response, evidenced by increased BMD in the tumor-injected tibia relative to the contralateral sham-operated control, 8–10 weeks post-inoculation. Within the defined ROI, VCaP- and 22Rv1-bearing bones demonstrated 22% (*n* = 17, *P* < 0.0001) and 19% (*n* = 12, *P* < 0.001) higher BMD, respectively, compared with controls. LNCaP-bearing bones showed a similar 27% increase (*n* = 11, *P* < 0.0001) (Fig. 1B).

To determine the contribution of adrenal-derived factors to the tumor-induced response of CRPC in bone, adrenals were removed in addition to ORX (ORX + ADX) before tumor cell inoculation (Fig 1A). The tumor-induced increase in BMD across all three models was nearly depleted in ORX + ADX mice compared with that observed in ORX mice: 100% (*P* < 0.0001) for VCaP-bearing bones, 85% (*P* < 0.0004) for 22Rv1-bearing bones, and 83% (*P* = 0.006) for LNCaP-bearing bones (Fig. 1C). This indicates that the tumor-induced sclerotic response in ORX mice is highly dependent on adrenal contribution, with the most pronounced effect observed in VCaP xenografts. Representative images of the tumor and sham-operated bones by μCT imaging after VCaP inoculation further supported the BMD results (Fig. 1D). The anti-sclerotic effect of ADX on BMD was exclusive to tumor-bearing bones, while BMD was slightly increased or similar in the sham-operated control bones (Fig. 1E). This suggests a protective effect of ADX on healthy non-tumor-bearing bones in ORX mice.

### Tumor-induced sclerotic bone response is associated with induced osteogenic and angiogenic processes in the bone

**Figure 2:**
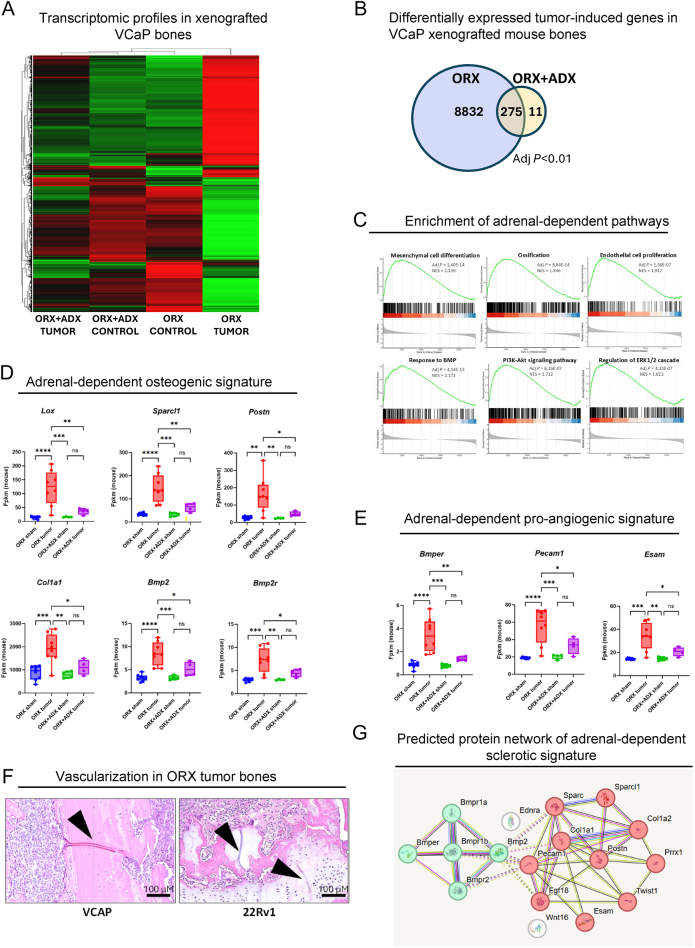
Adrenal regulation of the tumor-induced transcriptomic changes in the bone of VCaP-xenografted mice. Annotation of the RNA-seq data was carried out against the mouse reference sequence in tumor-bearing bones and sham-operated bones from VCaP xenografts. (A) Heatmap of unsupervised clustering of mouse genes (adj. *P* < 0.05) differentially expressed in bones with VCaP tumors and sham-injected control bones in ORX (*n* = 8) and ORX + ADX (*n* = 4) mice. (B) Venn diagram of mouse mRNAs (adj. *P* < 0.01) differentially expressed in tumor-bearing bones compared with the sham-operated control bones in ORX and ORX + ADX mice. (C) Gene set enrichment analysis of the pathways present in the tumor bones of the ORX mice that are highly altered in ORX + ADX mice. (D) Box plots of mRNA expression of genes related to osteogenic signature induced by the tumors in the ORX mice and suppressed in ORX + ADX mice. (E) Box plots of the key genes of the adrenal-dependent endothelial/pro-angiogenic signature induced by the VCaP tumors in the bones of ORX mice and downregulated in ORX + ADX mice. (F) Representative figures of tumor-induced vascularization, indicated by arrows, in VCaP and 22Rv1 tumor bones in ORX mice (H&E staining, 20× magnification). (G) String analysis of the transcriptomic data showing a predicted network of the key players in the adrenal-dependent sclerotic signature induced by the VCaP tumors in ORX mice. FPKM = Fragments per kilobase of transcript per million mapped reads. (D and E) The Kruskal–Wallis H test was used to determine differences between groups, *P* < 0.01 (**), *P* < 0.001 (***) and *P* < 0.0001 (****).

To investigate the role of adrenal contribution in the mechanisms underlying the tumor-induced bone response, we analyzed bulk RNA sequencing data annotated against the mouse (host) reference sequences from VCaP xenografted bones. VCaP cells were selected for the mechanistic transcriptomic analyses because they are widely recognized as one of the most clinically relevant models of human CRPC and exhibit the strongest ADX-dependent effect on the tumor-induced sclerotic bone response. The analysis included tumor-bearing and sham-operated bones of ORX (*n* = 8) and ORX + ADX mice (*n* = 4), and tumor-negative bones in the ORX + ADX group (*n* = 4) were excluded from the analysis. An unsupervised clustering analysis separated the expression profiles of tumor-bearing bones from ORX and ORX + ADX mice, which closely resembled those of sham-operated bones (Fig. 2A). Of all transcripts identified, 97% (8,832 transcripts) of differentially expressed (DE) genes (cutoff adj. *P* < 0.01) were exclusively altered in the tumor bones of the ORX mice, not being different in ORX + ADX tumor bones (Fig. 2B, Supplementary Table 1 (see section on Supplementary materials given at the end of the article)). In contrast, there were only 11 genes (<1% of all DE genes) whose expression was altered in the tumor bones of the ORX + ADX mice but were not different between ORX tumor bones and ORX sham bones. The tumor-induced response in both conditions shared 275 transcripts (3% of all DE genes, Fig. 2B). Thus, these genes are most likely not responsible for the sclerotic response observed in ORX mice.

A gene set enrichment analysis (GSEA) of the DE genes (adj. *P* < 0.05) between tumor-bearing bones in ORX and ORX + ADX mice revealed both previously known and novel pathways associated with sclerotic bone growth. The tumor-bearing bones of ORX mice, compared with ORX + ADX mice, exhibited the strongest enrichment of pathways related to mesenchymal stem cell differentiation, ossification, and cellular response to bone morphogenic protein (BMP) (Fig. 2C, Table 1), all directly associated with osteogenesis and likely contributing to the pronounced sclerotic phenotype. In addition, PI3K/Akt signaling and ERK1/2 signaling, together with endothelial cell proliferation and positive regulation of vascular development, were exclusively upregulated in the ORX tumor bones (Fig. 2C, Table 1), suggesting enhanced angio-osteogenic coupling that may further drive aberrant bone formation. In addition, focal adhesion, the Hippo signaling pathway, and endocrine processes were among the most downregulated pathways following ADX, indicating involvement of mechanotransduction and hormonal regulation.

**Table 1:** Gene set enrichment analysis of upregulated pathways in tumor-bearing bones from ORX mice compared with ORX + ADX mice.

ID	Description	NES*	*P*-value	Adj. *P-*value
GO:0030198	Extracellular matrix organization	2.333	1.986E-28	1.151E-25
GO:0060485	Mesenchyme development	2.169	1.101E-20	1.857E-18
GO:0001822	Kidney development	2.101	8.716E-19	8.399E-17
GO:0048762	Mesenchymal cell differentiation	2.140	1.870E-16	1.401E-14
GO:0001503	Ossification	1.946	1.442E-15	8.843E-14
GO:0071773	Cellular response to BMP stimulus	2.173	9.364E-15	4.141E-13
GO:0007409	Axonogenesis	1.845	9.042E-15	4.141E-13
GO:0030509	BMP signaling pathway	2.164	2.481E-13	7.605E-12
GO:0001837	Epithelial-to-mesenchymal transition	2.186	2.879E-13	8.630E-12
GO:0007160	Cell–matrix adhesion	2.040	3.535E-13	1.037E-11
GO:0001649	Osteoblast differentiation	1.915	1.193E-09	1.577E-08
GO:0016055	Wnt signaling pathway	1.674	3.489E-09	4.202E-08
GO:0071559	Response to transforming growth factor beta	1.857	3.645E-09	4.275E-08
GO:0030510	Regulation of BMP signaling pathway	2.072	4.328E-09	5.033E-08
GO:0045766	Positive regulation of angiogenesis	1.909	8.282E-09	9.166E-08
GO:1904018	Positive regulation of vasculature development	1.909	8.282E-09	9.166E-08
GO:0001935	Endothelial cell proliferation	1.918	1.496E-08	1.565E-07
GO:0070374	Positive regulation of ERK1 and ERK2 cascades	1.822	4.714E-08	4.326E-07
mmu04151	PI3K–Akt signaling pathway	1.712	2.568E-08	6.164E-07
mmu04510	Focal adhesion	1.776	3.190E-07	5.104E-06
mmu04390	Hippo signaling pathway	1.813	1.178E-06	1.616E-05
GO:0060349	Bone morphogenesis	1.858	2.998E-06	1.759E-05
GO:0045446	Endothelial cell differentiation	1.848	4.027E-06	2.302E-05
GO:0050886	Endocrine process	1.842	4.277E-06	2.394E-05
GO:0007219	Notch signaling pathway	1.654	2.878E-05	1.330E-04

Gene set enrichment pathway analysis (GSEA) of the mouse annotated differentially expressed genes (adj. *P* < 0.05) between tumor bones in orchiectomized (ORX) and ORX + adrenalectomized (ORX + ADX) mice (*n* = 8 and *n* = 4, respectively).

* NES, normalized enrichment score.

A unique osteogenic signature, including members of the BMP family (*Bmp2* and *Bmpr2*) and several genes related to BMP action, such as lysyl oxidase (*Lox*), periostin (*Postn*), SPARC-like 1 (*Sparcl1*), and collagen a1 and a2 (*Col1a1, Col1a2)*, was exclusively upregulated in tumoric bones of ORX mice compared with tumor-bearing bones from ADX mice and sham-operated control bones (Fig. 2D). In addition, several markers associated with vascular development and *de novo* angiogenesis were significantly upregulated in the tumor-bearing bones from ORX mice, including BMP-binding endothelial cell precursor-derived regulator *(Bmper)*, platelet endothelial adhesion molecule *(Pecam-1)*, and endothelial cell-selective adhesion molecule (*Esam*) (Fig. 2E). The presence of large blood vessels in areas connecting bone and tumor in VCaP and 22Rv1 xenografted bones in the ORX mice was confirmed by histological analysis and thus supports the transcriptomic data of adrenal regulation of tumor-induced vascularization (Fig. 2F). String analysis of the predicted protein network of the unique tumor-induced osteo-angiogenic signature in sclerotic ORX bones revealed two distinct functional clusters: BMP signaling and ECM-proteoglycans. The BMP signaling cluster underscores its pivotal role in osteogenic differentiation, while the ECM-proteoglycan cluster highlights the importance of modulating cell adhesion, growth factor availability, and angiogenic responses within the tumor-altered microenvironment (Fig. 2G). Altogether, the data indicate that adrenal contribution is critical for both the osteogenic and angiogenic processes in the bone compartment.

### Adrenal-dependent factors drive the CRPC tumor growth in the bone environment

**Figure 3:**
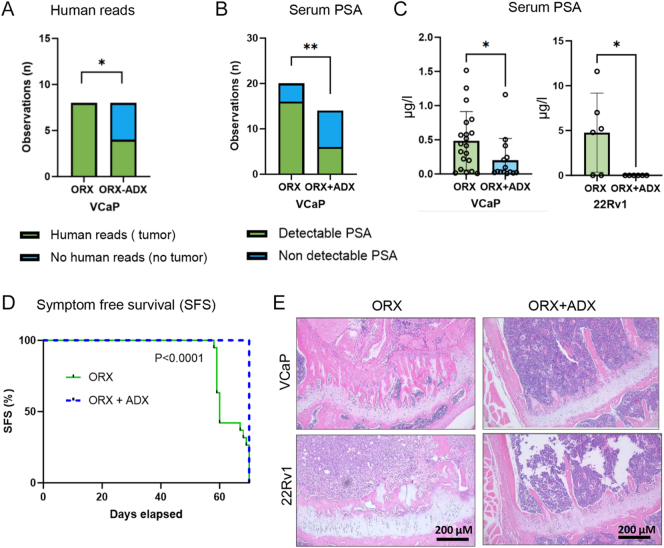
Growth of VCaP tumors in the bones of orchiectomized (ORX) and orchiectomized and adrenalectomized (ORX + ADX) mice. (A) Number of mice with a significant number of human reads in RNA sequencing data of the bones in ORX (*n* = 8) and ORX + ADX mice (*n* = 8), 10 weeks after VCaP cell inoculations: reads detected (green bar); reads not detected (blue bar). Five percent of human reads of the total number of reads was set as a cutoff for detected reads. (B) Number of animals with detectable (green bar) and non-detectable (blue bar) serum PSA levels in ORX and ORX + ADX mice with intratibial VCaP xenografts. (C) Serum PSA levels (μg/L) in mice at the time of termination in VCaP (*n* = 20) and 22Rv1 (*n* = 12) tibial xenografted ORX and ORX + ADX mice. (D) Kaplan–Meier curve of symptom-free survival (SFS) of VCaP xenografted ORX (*n* = 19) and ORX + ADX (*n* = 17) mice. (E) Representative images (10× magnification) of hematoxylin-and-eosin-stained tumor-bearing bones 10 weeks after VCaP and 22Rv1 inoculation in ORX and ORX + ADX mice. A chi-square test was used to determine the significance between human reads and tumor take (A) and between serum PSA and tumor take rate (B). A Mann–Whitney U test was used to compare differences between groups in (C). *P* < 0.05 (*) and *P* < 0.01 (**).

In addition to the targeted bone response, ADX reduced intratibial tumor growth of CRPC to approximately 50%, evidenced by the lack of human reads (<5% of total reads) in 4 out of 8 of the VCaP-injected bones of ORX + ADX mice, while a significant number of human reads were presented in all tumor-grafted bones of the ORX mice (*n* = 8) at the end of the 10-week-long follow-up period (Fig. 3A). In addition, using serum PSA as a marker of tumor burden, 80% of the ORX mice showed detectable serum PSA compared with only 40% of the ORX + ADX mice inoculated with the VCaP cells (*P* < 0.005) (Fig. 3B). Furthermore, the mean PSA in the serum of the mice with detectable VCaP tumors was >tenfold lower in the ORX + ADX mice compared with ORX only (Fig. 3C). Similar data were demonstrated with 22Rv1, while PSA levels were under the detection level in all of the ORX + ADX mice (Fig. 3C). The anti-tumor effect of ADX in ORX mice was also demonstrated by the degree of symptom-free survival (SFS) of the mice. At the termination, 10 weeks after VCaP tumor cell inoculation, 100% of the ORX + ADX (*n* = 16) had survived without adverse effects, in contrast to 26% SFS of the ORX mice that needed to be terminated due to the high tumor burden (median survival = 60 days, *n* = 19, *P* < 0.0001, Fig. 3D). The lack of a significant tumor in VCaP- and 22Rv1-injected bones in the ORX + ADX mice was confirmed by histological analysis (Fig. 3E).

### Adrenal-derived androgens are key drivers of the sclerotic CRPC tumor phenotype

**Figure 4:**
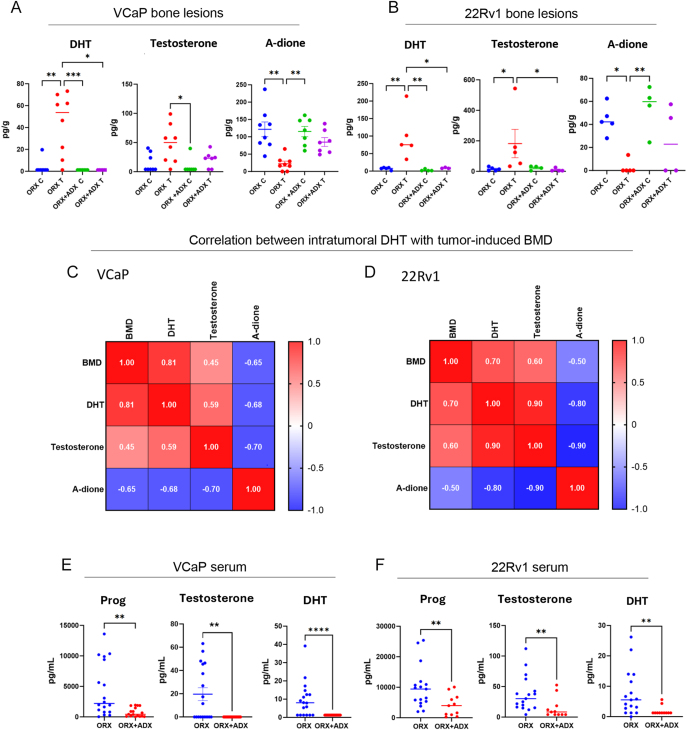
Intrabone and serum steroid concentration in orchiectomized (ORX) and orchiectomized and adrenalectomized (ORX + ADX) mice. (A and B) Scatter plots of dihydrotestosterone (DHT), testosterone, and androstenedione (A-dione) concentrations in tumor tibia (T) after VCaP (A) and 22Rv1 (B) cell inoculations, and in the sham-injected control (C) tibia of the same mice. (C and D) Spearman’s rank correlations between steroid levels in tumor-grafted bones and BMD induced by VCaP (*n* = 12) and 22Rv1 (*n* = 9) xenografts. Data were collected from both ORX and ORX + ADX mice. (E and F) Serum progesterone, DHT, and testosterone concentrations in mice after VCaP (C) and 22Rv1 (D) cell inoculations. Levels below the lowest level of quantification (LLOQ) were set to half of LLOQ. A Mann–Whitney U test was used to compare differences in serum levels between ORX mice and ORX + ADX mice. A Kruskal–Wallis H test was used to determine differences in intrabone levels between all groups. *P* < 0.05 (*), *P* < 0.01 (**), *P* < 0.001 (***), and *P* < 0.0001 (****).

When analyzing levels of androgens in the bone lesions of VCaP and 22Rv1 models, it became evident that high intratissue levels of DHT and testosterone were associated with the sclerotic response in the tumor-bearing bone, the values being >tenfold higher than in the sham-operated tibia of the same mice (*P* = 0.003 for VCaP and *P* = 0.006 for the 22Rv1). Furthermore, the DHT levels in the tumor-bearing bones in ORX mice were significantly higher compared with ORX + ADX mice in both VCaP and 22Rv1 xenograft models (Fig. 4A and B), and in the ORX + ADX mice, the values were at a similar level to those in the sham-operated bones of the ORX-treated mice. The testosterone levels followed a trend similar to DHT, with reduced levels in the tumoric bone of the ORX + ADX compared with ORX (for 22Rv, *P* = 0.013; for VCaP, *P* = 0.25). Notably, intratissue levels of androstenedione (A-dione), an immediate precursor of testosterone synthesis, were significantly lower in the tumor tibia in ORX mice compared with the control tibia of the ORX mice and in both tumor-bearing and sham-operated ORX + ADX mice. The role of the intratumoral DHT as a driver for the sclerotic response was further suggested by a significant correlation between the DHT levels and tumor-induced BMD (Fig. 4C and D) in both models (VCaP, *r*_s_ = 0.81, *P* = 0.003, *n* = 12; and 22Rv1, *r*_s_ = 0.71, *P* = 0.002, *n* = 9). In contrast, levels of A-dione were negatively correlated with both BMD and DHT, suggesting an induced conversion of A-dione to DHT as one of the key factors inducing bone growth in CRPC-mimicking conditions in our model.

To determine the role of adrenal-derived precursors for the local DHT concentration in the bone lesions, serum progesterone, testosterone, and DHT levels were measured (Fig. 4E and F). The median level of serum progesterone in VCaP-grafted ORX mice was 2,205 pg/mL, while ORX + ADX mice had a median level of 432 pg/mL (*P* = 0.002). Similar results (*P* = 0.017) were obtained in mice with 22Rv1 tumors. In addition, the serum concentration of the active androgens, testosterone and DHT, was markedly reduced and mainly below the detection limit of the assay by ADX of the ORX mice in both tumor models.

Furthermore, analysis revealed that 1,153 of the tumor-induced genes in the bone of ORX mice were significantly correlated with serum progesterone (*r*_s_ > 0.85, adj. *P* < 0.05), with 411 genes showing positive correlation and 742 genes showing negative correlation (Supplementary Table 2). In contrast, there were no significant correlations between serum testosterone or DHT and tumor-induced genes in the bones, suggesting their local production in the tumors. Collectively, the data indicate that tumor androgens derived from adrenal-produced progesterone are critical for the sclerotic progression of CRPC in bone, although progesterone itself or other adrenal factors may also contribute.

### Adrenal contribution is critical to maintaining AR signaling coupled to the energy metabolism of the tumor cells

**Figure 5:**
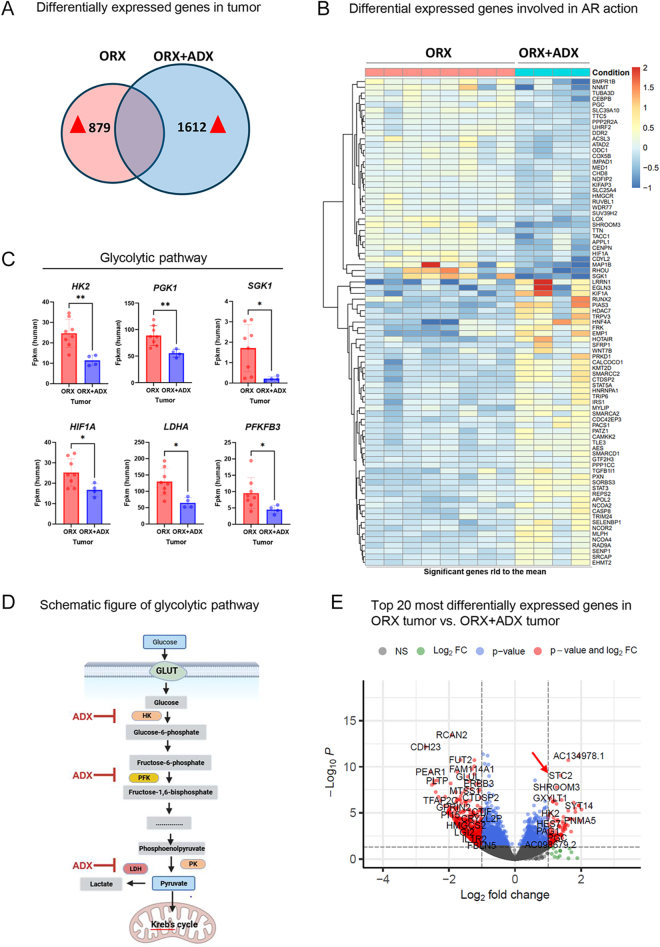
Differential mRNA expression in VCaP tumors from orchiectomized (ORX) and orchiectomized and adrenalectomized (ORX + ADX) mice. Annotation of the RNA-seq data was carried out against the human reference sequence. (A) Of the 37,455 human transcripts identified in the tumor bones, 2,491 genes were differentially expressed between ORX tumors and ORX + ADX tumors. Of these, the expression of 879 genes was higher in the tumors of the ORX mice, and for 1,612 genes, the expression was higher in the tumors of the ORX + ADX mice. The red arrowheads indicate the number of upregulated genes compared with the other conditions. (B) Hierarchical clustering of the 85 transcripts of proteins involved in AR action that showed differential expression between the tumors of ORX and ORX + ADX mice (adj. *P* < 0.05). (C) Scatter dot plots of the mRNAs of representative players in the glycolytic pathway and energy metabolism in the tumors of the ORX mice, downregulated by ADX in ORX mice. The Mann–Whitney U test was used to compare differences between groups; bars represent mean with SD, *P* < 0.05 (*), *P* < 0.01 (**). (D) Schematic figure of the adrenal-dependent steps in the glycolytic pathway in sclerotic bone lesions of VCaP xenografts in the ORX mice. (E) Volcano plot representing all the differentially expressed human transcripts (adj. *P* < 0.05) in the intratibial VCaP xenografts between ORX and ORX + ADX mice. The data highlight a high expression of *STC2* in the tumors in ORX mice, which is markedly downregulated in ORX + ADX mice.

Next, we annotated the RNA sequencing data against the human reference genome and compared the transcriptomic differences in the tumor between the ORX (*n* = 8) and ORX + ADX (*n* = 4) mice. The data revealed that the ORX tumors and ORX + ADX tumors were fully distinguished by unsupervised clustering, as demonstrated by a heatmap (Supplementary Fig. 1). Of the 37,455 human transcripts identified, 7% (2,491 genes, adj. *P* < 0.05) were differentially expressed between the cancer cells of the two conditions. Of these, the expression of 879 genes was higher in the tumors of the ORX mice, and for 1,612 genes, the expression was higher in the tumors of the ORX + ADX mice (adj. *P* < 0.05, Fig. 5A, Supplementary Table 3). These findings suggest that the ‘adrenal-resistant’ tumors are driven by distinct mechanisms compared with those responsible for the formation of sclerotic bone lesions observed in ORX mice. Consequently, the two conditions give rise to divergent bone metastatic phenotypes.

Enrichment analysis indicated that, among others, genes involved in central energy metabolism were differentially expressed in the tumors of ORX mice compared with those in ORX + ADX mice. The most enriched pathways in the tumor of the ORX mice included cellular respiration, ATP metabolic processes, and mitochondrial transmembrane transport (Supplementary Table 4), and the terms identified with the KEGG analysis included oxidative phosphorylation (OXPHOS), among others (Supplementary Table 4). This indicates that adrenal-dependent factors are essential for adapting tumor cells to the hypoxic/energy-demanding bone microenvironment.

In addition, 85 of the total 879 DE genes in the tumor cells in the ORX mice compared with ORX + ADX mice (adj. *P* < 0.05) showed an overlap with our literature-based gene list related to AR-signaling (Supplementary Table 5), and the tumors of ORX and ORX + ADX mice were also separated in a hierarchical clustering based on these genes (Fig. 5B). The androgen-dependency of the metabolic traits in the tumor compartment was further supported by higher expression of several AR-regulated genes of the glycolytic pathway in ORX tumors compared with ORX + ADT tumors. Those included hexokinase 2 (*HK2)*, which catalyzes the first irreversible step of the glycolytic pathway; phosphoglycerate kinase 1 *(PGK1)*; 6-phosphofructo-2-kinase *PFKFB3 (PFK2)*; and lactate dehydrogenase A *(LDHA)*, which converts pyruvate to lactate. In addition, serum and glucocorticoid-inducible kinase 1 *(SGK1)*, having a central role in glucose uptake, and hypoxia-inducible factor 1-alpha *(HIF1α)*, involved in the regulation of OXPHOS and glycolysis, were expressed higher in ORX tumors compared with ORX + ADX tumors (Fig. 5C). The adrenal-regulated glycolytic pathway of the tumors in ORX mice is illustrated in Fig. 5D.

Among the transcripts for secretory proteins expressed in the ORX tumors, we identified 13 transcripts strongly downregulated by ADX (log2 FC > 1, *adj. P* < 0.01, Table 2). Those at the top of the list included stanniocalcin 2 (*STC2*), followed by ependymin-related 1 (*EPNDR1*), glycoprotein hormones, alpha polypeptide (*GCA*), and growth differentiation factor 15 (*GDF15*) (Table 2; Fig. 5E). Accordingly, STC2 mRNA expression was exclusively restricted to the tumor compartment in the sclerotic bones with VCaP and 22Rv1 tumors, while the STC2 protein was detected in the tumor compartment and the surrounding osteoblasts and endothelial cells (Supplementary Fig. 2). Collectively, the data suggest that the sclerotic tumor phenotype is dependent on adrenal contribution.

**Table 2:** Differential expression of mRNAs encoding secreted proteins between ORX- and ORX + ADX-treated tumors.

Gene_name	BaseMean	Log2 FC	*P* value	Adj. *P-*value	Gene_description
*STC2*	283.61	1.38	6.74E-13	8.70E-10	Stanniocalcin 2
*EPNDR1*	531.97	1.26	3.27E-09	1.04E-06	Ependymin-related 1
*CGA*	29.46	2.02	1.92E-08	4.31E-06	Glycoprotein hormones, alpha polypeptide
*GDF15*	279.93	1.47	2.17E-07	2.76E-05	Growth differentiation factor 15
*GFOD1*	286.35	0.79	7.84E-07	6.88E-05	Glucose–fructose oxidoreductase domain-containing 1
*LPL*	418.49	1.54	2.53E-06	1.69E-04	Lipoprotein lipase
*CXCL13*	27.72	1.60	3.67E-06	2.29E-04	C–X–C motif chemokine ligand 13
*KLK15*	81.58	1.14	1.54E-05	6.64E-04	Kallikrein-related peptidase 15
*LOX*	77.14	1.00	7.86E-05	2.19E-03	Lysyl oxidase
*DEFB132*	7.50	1.42	9.51E-05	9.51E-05	Defensin beta 132

Differential expression of human annotated protein-coding genes of secreted proteins between tumors from orchiectomized (ORX) and ORX + adrenalectomized (ORX + ADX) mice (*n* = 8 and *n* = 4, respectively). Genes with a log_2_ fold change (FC) > 1 and an adjusted *P*-value <0.05 were considered significantly differentially expressed.

## Discussion

To our knowledge, this is the first study to demonstrate that adrenal-derived androgen precursors are critical for the development and maintenance of the sclerotic phenotype in metastatic CRPC. These findings provide a mechanistic explanation for how sclerotic lesions can be established and continue to progress despite ADT, revealing a previously unrecognized and essential role for the adrenal glands in driving sclerotic progression of CRPC within the bone microenvironment.

The primary endpoint of our study was tumor-induced sclerotic bone response. In our intratibial model, all AR-positive cell lines induced a broadly similar sclerotic bone response in ORX mice, with the most pronounced effect in LNCaP, which harbors a mutated, promiscuous AR with high affinity for adrenal androgen precursors such as pregnenolone and progesterone (38, 39). By contrast, VCaP and 22Rv1, which both express AR-variants (AR-Vs), exhibited a more pronounced response following ADX. Consistent with prior reports, AR-Vs are more frequently detected in bone metastasis, particularly in CRPC, than in hormone-naïve disease (40). Together, these observations suggest that AR-Vs may play a role in modulating the tumor–bone interactions and altering the bone response during advanced disease progression. Beyond attenuating the sclerotic bone phenotype, ADX significantly reduced tumor growth and serum PSA levels in tumor-bearing mice, indicating that both tumor expansion and tumor–bone crosstalk remain largely adrenal-dependent in sclerotic CRPC, despite castrate levels of circulating androgens. These findings align with our previous work with studies from other groups using subcutaneous CRPC models (54, 55). In contrast to the subcutaneous environment, bone is a highly organized organ with substantial cellular complexity, consisting of osteoblasts, osteoclasts, osteocytes, specialized endothelial cells, and diverse immune cell populations. Despite this complexity, ADX effectively reduced the CRPC growth in the bones. Notably, our data highlight adrenal-dependent intratumoral androgen synthesis as a key driver of both the sclerotic tumor phenotype and the tumor-induced sclerotic bone response.

Adrenal-dependent cues also regulated signaling pathways central to tumor-induced bone formation and remodeling, including BMP, ERK1/2, and PI3K/Akt. In ORX mice, the sclerotic bone transcriptome prominently featured mesenchyme development, osteogenesis/ossification, and angiogenesis/vascularization (including endothelial proliferation), consistent with established hallmarks of sclerotic PC bone metastasis (41). Importantly, our work demonstrates that these hallmark signatures were lost upon adrenal removal, thus supporting the conclusion that the sclerotic metastatic niche is fundamentally dependent on adrenal-derived factors.

In ORX mice, we identified a distinct osteo-angiogenic gene signature encompassing osteo-angiogenic coupling, including *Postn, Sparcl, Bmp2, Lox, Bmper,* and *Pecam1*, associated with mesenchyme development, osteogenesis, and angiogenesis (including endothelial proliferation). This profile aligns with the established hallmarks of sclerotic PC bone metastasis (41) and androgen-dependent stroma gene expression signatures associated with prognostic values for primary PC (42). Focal adhesion signaling and the Hippo pathway were among the most downregulated pathways in the ADX tumor-bearing bones. Given that focal adhesions facilitate pro-tumor metastatic matrix remodeling by mediating mechanotransduction through integrin-ECM interactions and cytoskeletal tension (43), their suppression suggests impaired tumor–bone communication. These findings indicate that the adrenal regulation of focal adhesion signaling contributes to the maintenance of the sclerotic metastatic bone niche. Collectively, our results show that these hallmark signatures are lost upon adrenal removal, demonstrating that both tumor–bone interactions and the maintenance of sclerotic metastatic bone niches are fundamentally dependent on adrenal-derived factors.

Alterations in mitochondrial energy metabolism and glycolysis are characteristic features of AR-driven traits in PC/CRPC tumors (44), and AR-mediated reprogramming promotes metabolic shift toward oxidative phosphorylation (45). Consistently, in our study, modeling the bone lesions of CRPC, OXPHOS and glycolysis signatures were among the most downregulated in the tumors from ORX + ADX compared with ORX mice. This coincided with a significant decrease in intratumoral DHT, suggesting AR-driven growth supported by high intratumoral DHT in ORX mice. The rapid growth and metastatic potential of tumors are closely linked to their enhanced glycolysis, glucose uptake rate, and glucose utilization (46, 47). The upregulation of key elements of the glycolytic pathway, such as *HK2, PFK,* and *LDHA,* indicates that the Warburg effect (a hallmark of cancer cells) is a characteristic phenotype also in the sclerotic CRPC bone lesions. The sclerotic tumor phenotype in our model mimics the most common subtype (BM1) from recent proteomic characterizations of human CRPC bone metastasis (48), marked by canonical AR target genes and similar metabolic traits to those of ORX mice in the present study. Notably, BM1 tumors displayed a higher degree of epithelial cell differentiation, evidenced by a high proportion of secretory cells (48). Likewise, mRNAs for secretory proteins were among the most significantly downregulated by ADX in the tumors. Among these, STC2 emerges as a novel and potential paracrine factor involved in the sclerotic response. It is a multifunctional protein involved in cell metabolism and calcium and phosphate uptake in different organs (49), implicated in the advancement of osteosarcoma, particularly in chondroblastic and osteoblastic phenotypes (50). Furthermore, high *STC2* expression correlates with a high Gleason score and metastasis (51) and promotes osteogenic differentiation of bone marrow mesenchymal stem cells, partly by inhibiting adipogenesis via PI3K/AKT and ERK signaling pathways (52, 53). These observations warrant further evaluation of STC2 in sclerotic CRPC, particularly in osteo-angiogenic coupling.

Serum progesterone, testosterone, and DHT were all markedly reduced following ADX in the intratibial tumor-grafted ORX mice, with the most pronounced decrease observed in progesterone. This systemic decline was accompanied by reduced intratumoral testosterone and DHT levels. These results align with previous studies in orthotopic and subcutaneous CRPC models, by us and others, showing that combined ADX and ORX significantly lower serum progesterone and DHT compared with ORX alone (54, 55). The data suggest that progesterone may act as a precursor for intratumoral A-dione synthesis, which is further utilized for local DHT production in CRPC tumors. Reduced A-dione levels in tumor-bearing bones relative to sham-operated controls support active conversion during local androgen synthesis in bone lesions. In ORX + ADX mice, the accumulation of intratumoral A-dione alongside decreased testosterone and DHT levels indicates a disruption in the conversion of A-dione to testosterone. We have previously reported that serum testosterone and DHT concentrations in non-tumor-bearing ORX male mice are below 4 and 2 pg/mL, respectively (29). Therefore, the elevated androgen levels observed in tumor-bearing mice in the current study are likely tumor-derived, a conclusion further supported by increased testosterone and DHT concentrations in tumor-bearing bones compared with sham-operated controls.

While DHEA-S is considered the predominant circulating adrenal precursor for peripheral androgen synthesis in men (56), the role of residual circulating progesterone in tumor progression remains unclear. In male mice, progesterone is predominantly of adrenal origin (57) and was similarly reduced following ADX in our model. Our findings implicate progesterone as a potential contributor to the progression of CRPC in bone, as evidenced by its association with sclerotic bone responses in our model. In fact, more than 10% of the tumor-induced genes upregulated in sclerotic ORX bones correlated with serum progesterone. These included regulators of osteogenesis and angiogenesis and focal adhesion (e.g. *Bmp2, Pdgfb*, and *Vim*) and metastasis-linked genes (*Met)* (Supplementary Table 2), several previously implicated in PC bone metastasis (41, 58, 59) and in progesterone-mediated effects on bone mesenchymal cells (60). Clinically, elevated serum progesterone has been associated with poor prognosis in CRPC (61) and has been proposed as a predictive biomarker for abiraterone response (61). Beyond serving as a steroid precursor, progesterone itself has been implicated as an oncogenic factor in CRPC (61), suggesting that inhibiting its synthesis could be a potential therapeutic target.

One emerging therapeutic strategy for metastatic CRPC is the use of CYP11A1 inhibitors, with one such compound, opevesostat, currently in extensive clinical trials (62). Similar to ADX, CYP11A1 inhibition blocks all steroid synthesis from the adrenals by preventing the conversion of cholesterol to pregnenolone, thereby blocking the formation of androgen precursors, glucocorticoids, and mineralocorticoids (63, 64). Ongoing clinical trials are evaluating opevesostat in mCRPC patients receiving ADT together with glucocorticoid/mineralocorticoid support, with rPFS as the primary study endpoint. The available data indicate their potential effect in patients with both wild-type AR-expressing tumors and those expressing a mutation in the ligand-binding domain of AR, supporting its potential as a comprehensive suppressor of AR signaling through the inhibition of DHT and other ligands (62). Historically, ADX was reported to delay disease relapse and improve radiographic regression of sclerotic bone metastases, although severe complications limited its clinical use (22, 23, 24). In contrast, CYP11A1 inhibition appears to offer a more favorable safety profile when combined with adjunctive hormonal therapy (62).

In conclusion, our findings demonstrate that adrenal-derived factors play a critical role in driving the sclerotic bone response in the CRPC setting. The intratumoral levels of active androgens, the metabolic characteristics of the tumor cells, and tumor–bone interactions all depend on adrenal contribution. Precursors for intratumoral androgen synthesis, such as progesterone, are likely involved, although other factors may also contribute (65). Our findings suggest that a comprehensive blockade of adrenal contribution is essential to prevent the sclerotic bone response associated with CRPC. The current data also highlight the importance of continuing clinical studies involving CYP11A1 inhibitors (62) in combination with ADT to pharmaceutically block all steroid synthesis from the adrenals.

## Supplementary materials















## Declaration of interest

All authors declare that they have no known competing financial interests or personal conflicts of interest that could have appeared to influence the work reported in this paper.

## Funding

This work was supported by the Swedish Research Council (Grant No.: VR 2020-01836, MP), the Swedish Cancer Foundation (Grant No.: 211889 Pj, MP), the Swedish Prostate Cancer Foundation (Grant Nos: 2020, 2022, MHT), and Assar Gabrielson’s foundation for preclinical research (Grant Nos: FB21-93, FB22-105, FB23-103, MHT). This work was also supported by grants from the Swedish state under the agreement between the Swedish government and the county councils (the ALF agreement; Grant No.: ALFGBG-965821, MP) and Cancer Foundation Finland (MP).

## Author contribution statement

MHT and MP conceived and designed the project. MHT, AL, LL, KH, and JW acquired the data. SA performed statistics and bioinformatic analysis of the RNA-seq data. MHT, SA, and AL analyzed the data. MHT, MP, and CO interpreted the data. MHT, CO, and MP drafted the manuscript. MHT, CO, KH, M-KL, LL, and MP revised the manuscript. All authors read and approved the final version of the manuscript.

## Data availability

The RNA-seq data have been deposited in the Gene Expression Omnibus (GEO) repository (accession code: GSE333317) and are publicly available. All other data supporting the findings of this study are included in the article and its supplementary materials.

## Ethical approval

All animal work was conducted in accordance with all relevant legislation and approved by the Ethical Committee for Animal Research in Gothenburg, Västra Götaland, Sweden (ethical permission no. 2476/19).

## Consent for publication

All authors approved the final version of the manuscript and agreed to its publication.
