# Supplementary material for: Adrenal-derived factors drive progression of sclerotic prostate cancer in bone
Source: Endocr Relat Cancer. 2026 Jun 9;33(6):e250309. doi: 10.1530/ERC-25-0309 (PMC13261362; doi:10.1530/ERC-25-0309)
Supplement: Supplementary file 1 [file supplementary_figure_1.pdf]

Supplementary Figure 1. Differentially expressed genes in ORX tumor vs. ORX+ADX tumor of VCaP xenografted mice.

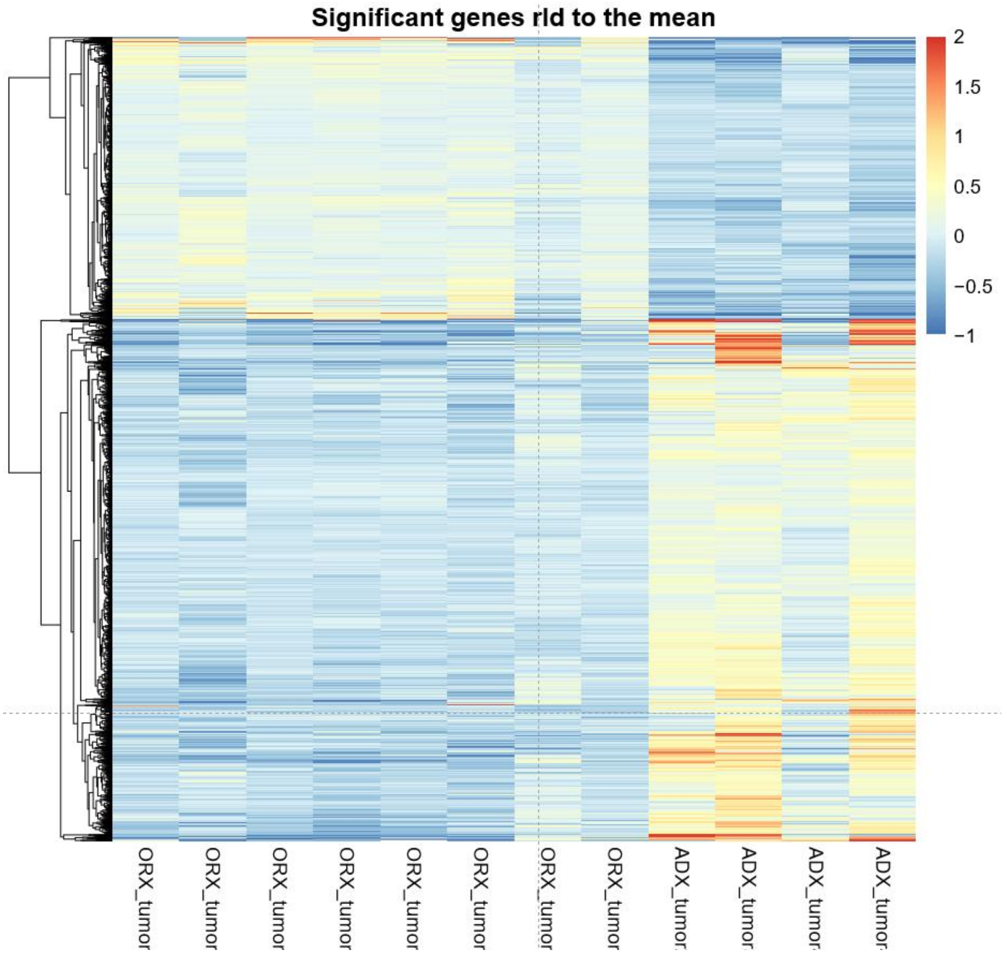

Unsupervised clustering of differentially expressed genes (human annotation, adj $p<0.05$) in intratibial xenografted VCaP tumors in orchiectomized ORX (n=8) vs ORX + adrenalectomized mice (ADX, n=4), visualized by a heatmap.
