# Supplementary material for: Adrenal-derived factors drive progression of sclerotic prostate cancer in bone
Source: Endocr Relat Cancer. 2026 Jun 9;33(6):e250309. doi: 10.1530/ERC-25-0309 (PMC13261362; doi:10.1530/ERC-25-0309)
Supplement: Supplementary file 2 [file supplementary_figure_2.pdf]

Supplementary Figure 2.

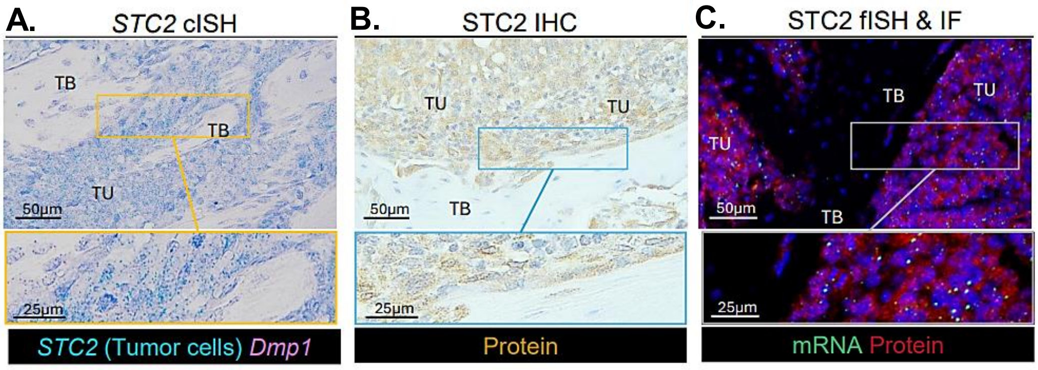

Representative figures of STC2 mRNA and protein expression in sclerotic tumor bone established in ORX mice with 22RV1 cells. A) Chromogenic in situ hybridization (cISH) of human STC2 mRNA and mouse Dmp1 mRNA in 22Rv1 xenografted bone lesion in ORX mice, indicating the expression STC2 mRNA in the cancer cells. The mouse Dmp1 mRNA was used for localization in osteocytes to enhance staining interpretation. **B)** Chromogenic immunohistochemistry (IHC) revealed the presence of STC2 protein in the bone lesions of 22Rv1 cells. Co-localization of STC2 mRNA with fluorescence in situ hybridization (fISH, indicated in green) and immunofluorescence staining (IF) of STC2 protein (indicated in red) is depicted, with nuclear staining (DAPI) is shown in blue. Abbreviations: TB= trabecular bone, TU= tumor.

Methods

In situ hybridization

The chromogenic in situ hybridization (cISH) and fluorescent in situ hybridization (fISH) on FFPE tumor-injected bones with VCaP and 22Rv1 cells in ORX (n=3+3) mice were performed by using the RNAscope Duplex Detection Kit (ACD; 322500) and the Multiplex Fluorescent Detection Reagent v2 (ACD; 323110). Standard RNAscope protocols were applied according to the manufacturer's instructions, with minor modifications described below. Rehydrated sections were blocked by hydrogen peroxide and heated in target retrieval buffer, then digested by 10% pepsin. Target probes were then applied and incubated overnight at 40°C. The following probes were used: Hs-STC2-No-XMm (ACD; 240541-C1), Mm-Dmp1 (ACD; 441171-C2). After 10 rounds of amplification, chromogenic signals were generated using a kit provided by the manufacturer for fast red (C2 channel) and green (C1 channel) dyes. Sections were subsequently counterstained with 50% hematoxylin. The fluorescent signal was visualized using the TSA Plus Cyanine 3 system (PerkinElmer, NEL744001KT). Images were captured using either a Nikon Eclipse 80i microscope or a Nikon Spinning Disk system.

Immunohistochemistry

Immediately following STC2 RNAscope detection for fISH, immunofluorescence (IF) was carried out on the same section. Blocking serum was added and allowed to incubate for 1 hour before applying the primary antibody against STC2 (Sigma-Aldrich, HPA045372), which was diluted 1:200 in blocking serum and then left to incubate overnight at 4°C. The secondary antibody (Jackson Immunoresearch, 711-605-152) was then applied and incubated for 1 hour at room temperature, followed by counterstaining with DAPI. Images were taken by a Nikon Spinning Disk system. A chromogenic immunohistochemistry (IHC) was also performed for STC2 using the primary antibody described above. After the initial incubation, sections were treated with a biotinylated secondary antibody (BP-9100-50, goat anti-rabbit, Vectastain) for 1 hour, followed by the ABC-HRP Kit (PK-6100, Vectastain) for another hour, according to the manufacturer's instructions. The Liquid DAB substrate chromogen system (K3468, Dako) was used as the peroxidase substrate for enzymatic amplification, and hematoxylin was applied for counterstaining. After dehydration, the slides were mounted using Pertex (00871.0500, Histolab).
